# Analysis of the sources of uncertainty for EDR2 film‐based IMRT quality assurance

**DOI:** 10.1120/jacmp.v7i2.2230

**Published:** 2006-05-25

**Authors:** Chengyu Shi, Nikos Papanikolaou, Yulong Yan, Xuejun Weng, gyu Jiang

**Affiliations:** ^1^ Cancer Therapy and Research Center and UTHSCSA 7979 Wurzbach San Antonio Texas 78229; ^2^ Department of Radiation Oncology University of Arkansas for Medical Sciences 4301 W. Markham Street Little Rock Arkansas 72202 U.S.A.

**Keywords:** IMRT, EDR2, calibration curve, uncertainty, film dosimetry

## Abstract

In our institution, patient‐specific quality assurance (QA) for intensity‐modulated radiation therapy (IMRT) is usually performed by measuring the dose to a point using an ion chamber and by measuring the dose to a plane using film. In order to perform absolute dose comparison measurements using film, an accurate calibration curve should be used. In this paper, we investigate the film response curve uncertainty factors, including film batch differences, film processor temperature effect, film digitization, and treatment unit. In addition, we reviewed 50 patient‐specific IMRT QA procedures performed in our institution in order to quantify the sources of error in film‐based dosimetry. Our study showed that the EDR2 film dosimetry can be done with less than 3% uncertainty. The EDR2 film response was not affected by the choice of treatment unit provided the nominal energy was the same. This investigation of the different sources of uncertainties in the film calibration procedure can provide a better understanding of the film‐based dosimetry and can improve quality control for IMRT QA.

PACS numbers: 87.86.Cd, 87.53.Xd, 87.57.Nk

## I. INTRODUCTION

Intensity‐modulated radiotherapy (IMRT) is a highly conformal treatment modality that requires precise verification. In our facility, patient‐specific IMRT quality assurance is routinely performed for each patient. A point dose measurement is obtained using a 0.125 cm^3^ ion chamber (PTW‐FREIBURG, Freiburg, Germany), and a coronal planar dose is measured using Kodak EDR2 film (Eastman Kodak Company Rochester, NY). With an ion chamber one typically measures a single point at a time; therefore, it is desirable to have 2D dose comparison between a film measurement and the treatment‐planning prediction. In order to obtain accurate absolute dose from the film, a dose calibration curve has to be accurately established. The film calibration curve can be affected by several factors, such as film batch differences, film processor temperature, and film digitization errors. For these reasons, we have elected to derive a film calibration curve every time film dosimetry is performed.

The EDR2 film allows for a wide, dynamic range of doses (25 cGy to 400 cGy), which is especially useful in routine patient IMRT QA. In addition, it is relatively insensitive to X‐ray energy selection and is easy to process. Zhu et al.^(1)^ evaluated EDR2 film for static multileaf collimator IMRT dose verification in high‐energy photon beams and concluded that EDR2 film is less sensitive than XV film. EDR2 film sensitivity was found to be independent of dose rate or dose per pulse. Field size and depth had little effect on the calibration curves.^(1)^ In their comparison between ion chamber measurements and film, they found a 2.5% agreement.^(1)^ It was also shown that the calibration curve cannot be approximated using a linear function. Gerbi and Dimitroyannis^(2)^ studied the response of EDR2 film in high‐energy electron beams. Their study showed that EDR2 film exhibits an energy‐dependent enhancement for electron beams and that the response of EDR2 film increased with increasing electron energy. Dogan et al.^(3)^ compared EDR2 and XV2 films for the verification of IMRT and found that EDR2 deviated from ion chamber for field sizes larger than 24cm×24cm. Childress et al.^(4)^ also verified IMRT dosimetric accuracy using EDR2 film and indicated that EDR2 film may underestimate doses for prolonged delivery times. They also concluded that the daily film calibrations are necessary since the standard deviations of optical density were between 7% and 15% for the averaged calibration curve over a period of 18 months. Morrell and Rogers^(5)^ used EDR2 film in cardiac catheterization procedures for patient skin dose assessment and summarized that for the diagnostic energy range, the EDR2 film is relatively insensitive to batch differences, field size, exposure rate, time to processing, and day‐to‐day fluctuations in processor performance.

In our investigation, we developed a more theoretical analysis of the sources of uncertainty in film dosimetry with the EDR2 film that complements the work that is published in the literature.

## II. METHODS

### A. Experiment facilities

In our institution, we have two Varian 2100EX linear accelerators and one HiArt tomotherapy unit. The tomotherapy unit produces a 6‐MV beam, while the Varian units have dual energy capability of 6 MV and 18 MV. Most of our IMRT planning is done based on 6‐MV beams.

The film processor used in this study was a Kodak X‐OMAT 3000RA. The processor operating characteristics are programmable. The chemistry is replenished every month. The developer, fixer, and dryer temperatures are preset to 37.2 °C (99° F), 37.2 °C (99° F), and 48.9 °C (120° F), respectively, and the processor is digitally controlled. The user can manually change the processor temperature and override the automatically maintained settings.

The films used in this study were scanned using the VXR‐16 plus Dosimetry Pro film digitizer by Vidar Company (Herndon, VA). The normal resolution setting for IMRT QA is 2k×2.5k with 178 μm spot size, 142.5 dots per inch, and 2.8 line pairs/millimeter. The RIT 113 (v.4.1) software (Radiological Imaging Technology, Inc. Colorado, CO) was used for the film analysis. The RIT 113 version 4.1 forces the scanner to a self‐calibration anytime a new scan is performed. The film scanner can be calibrated using the National Institute of Standards and Technology (NIST) reference film with 33 discrete density steps and optical density ranging from 0.07 to 3.78. The calibration enables the conversion of scanner numbers to optical density. By default, a 3×3 median filter was used to filter the scanned films.

In order to obtain the calibration curve for a patient‐specific IMRT QA film, we developed two treatment unit—specific procedures. For the linear accelerator, we delivered a step wedge pattern with 13 incremental dose levels; each step is 2.0cm×10cm at the isocenter. For the tomotherapy unit, we delivered a step valley pattern that contains 11 in‐dispersed dose steps measuring 2.5cm×5cm each at the isocenter.^(7)^ The step wedge and step valley patterns are generated using the respective unit's multileaf collimator (MLC) and an appropriate MLC control file. The absolute dose (*D*) corresponding to each step was measured using a calibrated ion chamber. All the films were scanned using a Vidar film scanner, and the scanner number value (*S*) was associated with the corresponding measured dose to each step. The value of *S* corresponds to the light transmission (analog/digital conversion) of the film when scanned and can be converted to the film's optical density using the digitizer calibration curve if necessary. In this study, we used the scanner number value (*S*) instead of optical density. When the scanner value is plotted against dose, it provides a calibration or response curve. We chose to use scanner number instead of optical density because it is more sensitive to the gray value changes in the film as compared to optical density.

### B. Experiment setup

#### B.1 Film batch study

Two EDR2 films from each of the three boxes that were used were sampled for the batch study. The films were irradiated on the 2100EX unit with a 6‐MV photon beam. The step wedge pattern was used, and the film was placed at the isocenter with 6 cm of solid water phantom (Gammex RMI, Middleton, WI) as buildup material. The back scattering material was 10 cm of solid water. Each film was placed in the coronal plane with source‐to‐film distance of 100 cm. The films were exposed one by one and were put in a dark room after exposure. Once all six films were exposed, they were processed, developed, and digitized (scanned) sequentially.

#### B.2 Film processor temperature study

Six films from the same batch were sampled and irradiated using the step wedge pattern as was done previously with the film batch study. The film processor was operated at temperatures of 26.7 °C, 29.4 °C, 32.2 °C, 35.6 °C, and 37.2 °C (80° F, 85° F, 90° F, 96° F, and 99° F) for each film. Two films were processed at the temperature 37.2 °C (99° F) as reference films. After all the films were processed, each film was digitized.

#### B.3 Film digitizer study

An NIST calibration film was used to calibrate the film digitizer. Since the calibration film is smaller than the digitizer scanning area, different parts of the scanning area (left, middle, right) were used for the calibration. In addition, the calibration film was scanned on different days for the same scanning area to explore possible daily drifts.

#### B.4 Treatment unit study

The 6‐MV beam of the 2100EX and the tomotherapy units were compared by means of analyzing the dose response obtained from the step wedge and step valley films that we produced from those units over a period of one year. The calibration dose‐response curves were collected and averaged. Eighty‐three step wedge calibration curves and 60 step valley calibration curves were analyzed for this study.

#### B.5 Patient‐specific IMRT based on film study

Fifty patient‐specific IMRT QA plans were randomly selected to compare the differences between film‐based measurement, ion chamber measurement, and planned dose. Patient‐specific IMRT plans were developed using the Philips Pinnacle version 7.4 radiation treatment‐planning station, and the films were irradiated using the Varian 2100EX linear accelerator. No tomotherapy plans were included in this analysis. All the calibration and IMRT QA films were chosen from the same film batch, and the processing temperature was set to 37.2 °C (99° F). The films were scanned at the same position on the film digitizer (left position). The treatment sites included brain, head and neck, lung, abdomen, pelvis, and extremities. The measurement point with ion chamber was selected inside the treatment target with homogeneous dose distribution.

## III. RESULTS AND DISCUSSION

**Figure 1:**
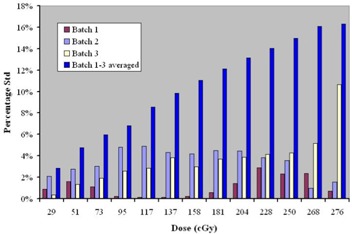
Percentage standard deviation versus dose value for batch study of EDR2 film

The magnitude of the differences in film manufacturing was evaluated by developing films from different film batches. Figure 1 shows the results of the film batch study for the EDR2 film. The *x*‐axis represents the absolute dose given (as measured with an ion chamber) to each step for the step wedge pattern that was executed on the Varian machine. The *y*‐axis shows the percentage standard deviation (Std) for the scanner number that was obtained by scanning the film with the Vidar scanner. Even though the percentage Std varies from batch to batch, the averaged percentage Std shows an increasing trend with increasing dose; the highest percentage Std is 16% for the dose step of 276 cGy. For the typical IMRT dose per fraction in the 180 cGy to 220 cGy range, it is preferable to use films from the same box, thus reducing the error in scanner response to less than 5%.

**Figure 2:**
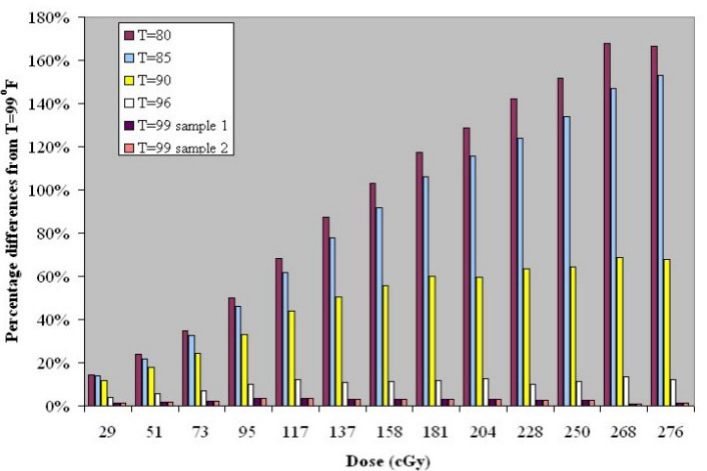
Percentage differences for scanner number for different film‐processing temperatures

The results of the study on the effect of temperature in film processing are shown in Fig. 2. The temperature of 37.2 °C (99° F) was used as the reference temperature, which is our preset operating temperature for the Kodak developer. The percentage difference from the reference temperature (37.2 °C) was plotted against the dose for each step. There are two samples for T=37.2 °C (99° F) and one sample for all other processing temperatures. The percent difference in scanner number increases with decreasing temperature and with increasing dose value. For T=37.2 °C (99° F), the percentage differences are less than 3%. The film processor is digitally controlled, and the processing temperature varies less than 0.5° F when the processor is in “ready” status. Therefore, the scanner number percentage differences will be less than 3% if the calibration film is processed when the processor is ready.

**Figure 3:**
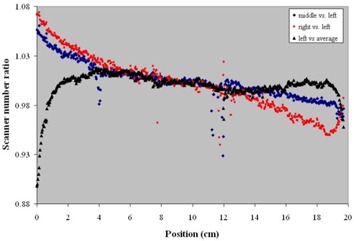
Film scanner number percentage changing with different scanning position

The effect of the scanning position and placement of the film on the scanner is shown in Fig. 3. Although we typically use the left side of the scanner to place the film against the film alignment feeder, we found that the middle and right placement positions result in different scanning numbers. This is likely due to differential response of the scanner detectors. Omitting the boundary response at the edge of the film, for the left‐aligned scanning position the difference in the scanning number for a constant film density is up to 3%. The difference between the middle and right scanning positions can be as high as 7%. Since all our IMRT QA films are scanned left‐aligned, one can conclude that we would observe no more than 3% variability in the scanner number.

**Figure 4:**
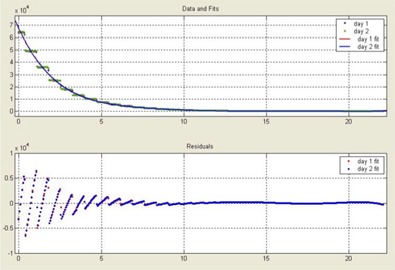
Film digitizer daily shift

The day‐to‐day variation in scanner number is plotted in Fig. 4. The curves are generated by scanning the calibrated NIST film in two separate days. The *x*‐axis represents the detector position in centimeters, and the *y*‐axis shows the scanner number. We used the MATLAB (v.6.5.1, The MathWorks Inc., Natick, MA) curve‐fitting tool “cftool” to generate a sixth‐degree polynomial that fits the data with 95% confidence level. The fitting curves for day 1 and day 2 overlap each other, which indicates that there is much less daily shift in the response of the scanner. The scanner number corresponding to 0 cm is a derived number estimated by the fitting curve. For the VXR‐16 scanner, this number should be 65535. The bottom curve shows the residual distribution for the fitting. The residuals are defined as the difference between measured data and fitted data. If data fitting is correct, the residuals approximate the random errors around zero. In other words, a random appearance of residuals suggests that the model fits the data well. From the residual curves we can see that the residual behaved randomly and overlap each other. Thus, the fitting model is correct with 95% confidence.

**Figure 5:**
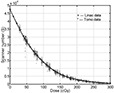
Scanner number versus dose for calibration curve over a period of one year

We have collected and analyzed the film calibration dose‐response curves for the linear accelerator and tomotherapy units over a period of one year. A plot of the data is shown in Fig. 5. By minimizing the least square distance to each data point, two fitting curves were derived: one for the linear accelerator and one for the tomotherapy unit. The linear accelerator and tomotherapy fitting curves are almost identical, which implies that the EDR2 film is virtually independent of the radiation unit. The fitting curves can be approximated by the following equation:

(1) D=Alog⁡S+B,

where *D* represents dose in units of centigrays, and *S* represents the scanner number. *A* and *B* are fitting coefficients, with A=−67.72cGy and B=741.6cGy. By taking the partial derivative of Eq. (1), the following formula is obtained:

(2) $$\frac{\partial D}{D}=\left(\frac{A}{A\log S+B}\right)\frac{\partial S}{S}=\left(\frac{1}{\log S+\frac{B}{A}}\right)\frac{\partial S}{S}=\left(\frac{1}{\log S-10.95}\right)\frac{\partial S}{S}.$$

Equation (2) shows the relationship between the dose variation $\frac{\partial D}{D}$ and the uncertainty in the scanner number $\frac{\partial S}{S}$. According to Eq. (2), for a typical dose D=200cGy, the value for *S* will be $2.97\times 10^{3}$. The dose and scanner uncertainties are then related by

(3) $$7\frac{\partial D}{D}\approx -\frac{\partial S}{S},$$

which means that a 1% change in dose will result in a 7% change in scanner number and vice versa.

By maintaining a consistent film calibration process, we can ensure that the percentage differences for scanner number will not exceed 5% for film batch factor, 3% for film processor temperature factor, 3% for film digitizer factor, and 3% for other unknown factors (such as setup uncertainty). If all of those factors are independent and follow a Gaussian distribution, the overall uncertainty for the scanner number will be

(4) $$\frac{\partial S}{S}=\sqrt{3{\%}^{2}+3{\%}^{2}+3{\%}^{2}+5{\%}^{2}}\approx 7{\%}^{2}.$$

According to Eq. (3), the uncertainty for dose D=200cGy will be about 1%. Including the daily linear accelerator output uncertainty of 2%, the typical IMRT QA uncertainty for dose range (180cGy~220cGy) will be less than 3% based on film dosimetry.

**Figure 6:**
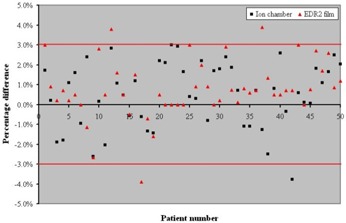
Distribution of patient‐specific IMRT QA dose percentage difference between ion chamber and EDR2 film

Fifty patient‐specific IMRT QA plans were analyzed based on ion chamber measurement and film measurement comparisons as shown in Fig. 6. All the IMRT QA cases were treated using a Varian 2100EX linear accelerator. The dose reading for EDR2 film was sampled in a point of high‐dose (>150cGy) and low‐dose gradient. Based on this analysis, we found the percent difference between the two measurement methods to be less than 3% for 94% of the cases studied. This outcome was achieved by controlling our film calibration process as described earlier.

## IV. CONCLUSIONS

EDR2 film is used for patient‐specific IMRT QA for 2D isodose distribution verification in several radiotherapy centers. Even though it is used mostly for relative dosimetry, it can also be used for absolute dose measurements provided the appropriate calibration process is followed. In this work, we investigated the sources of uncertainties that contribute to the daily variation of film response in film dosimetry. Appropriate selection of the parameters that contribute to the film dosimetry uncertainty can result in an overall uncertainty of up to 3% for the EDR2 film. We found that the uncertainty for the EDR2 film dosimetry was independent of the treatment unit. An analysis of a random sample of patient‐specific IMRT QA performed in our institution showed that with our technique, we can obtain a maximum of 3% uncertainty for the majority of our film‐based dosimetry.
